# Cholera-Like Enterotoxins and Regulatory T cells

**DOI:** 10.3390/toxins2071774

**Published:** 2010-07-06

**Authors:** Christelle Basset, Fatou Thiam, Cyrille Di Martino, John Holton, John D. Clements, Evelyne Kohli

**Affiliations:** 1 Laboratoire des Interactions Muqueuses-Agents transmissibles (LIMA), UPR562, UFRs Médecine et Pharmacie, IFR Santé-STIC, Université de Bourgogne, Dijon, France; Email: christelle.basset@u-bourgogne.fr (C.B.); thiam.fatou@hotmail.fr (F.T.); cyrilledimartino@yahoo.fr (C.D.M); 2 Windeyer Institute of Medical Sciences, University College London, London, UK; Email: john.holton@uclh.nhs.uk (J.H.); 3 Department of Microbiology and Immunology, Tulane University Health Sciences Center, New Orleans, LA 70112, USA; Email: jclemen@tulane.edu (J.D.C.)

**Keywords:** cholera-like enterotoxins, regulatory T cells, cholera toxin, heat-labile enterotoxin of *E. coli*, CTB, LTB

## Abstract

Cholera toxin (CT) and the heat-labile enterotoxin of *E. coli* (LT), as well as their non toxic mutants, are potent mucosal adjuvants of immunization eliciting mucosal and systemic responses against unrelated co-administered antigens in experimental models and in humans (non toxic mutants). These enterotoxins are composed of two subunits, the A subunit, responsible for an ADP-ribosyl transferase activity and the B subunit, responsible for cell binding. Paradoxically, whereas the whole toxins have adjuvant properties, the B subunits of CT (CTB) and of LT (LTB) have been shown to induce antigen specific tolerance when administered mucosally with antigens in experimental models as well as, recently, in humans, making them an attractive strategy to prevent or treat autoimmune or allergic disorders. Immunomodulation is a complex process involving many cell types notably antigen presenting cells and regulatory T cells (Tregs). In this review, we focus on Treg cells and cholera-like enterotoxins and their non toxic derivates, with regard to subtype, *in vivo/in vitro* effects and possible role in the modulation of immune responses to coadministered antigens.

## 1. Introduction

Cholera toxin (CT) from *Vibrio cholerae* and the related heat-labile enterotoxin (LT) from toxinogenic strains of *E. coli* are both A: B5 ADP-ribosylating exotoxins that cause abundant secretory diarrhea and enhance bacterial pathogenicity. They are also extremely potent immunogens and mucosal and parenteral adjuvants of immunization that potentiate mucosal and systemic B and T cell responses against unrelated co-administered antigens (Ags) in many experimental models (reviewed in [1,2,3,4,5]). Both toxins comprise a single A subunit responsible for the ADP-ribosyl transferase activity and five identical B subunits, responsible for cell binding. Another group of enterotoxins that are expressed by *E. coli* strains and have the same structure as CT and LT includes LT-IIa and LT-IIb (type II subfamily, CT and LT representing the type I subfamily) [6,7]. To overcome the enterotoxicity and use them as adjuvant in humans, non toxic mutants of the A subunit that retain adjuvant properties have been developed [1,2,3,4,5] and in some cases tested in clinical trials [8,9,10,11,12]. 

The B subunit of these toxins is devoid of toxicity and thus could also be used as adjuvants in humans. However, the adjuvant activity of the B subunit has been early a source of controversy and the question remains confusing. Moreover, the B subunits of CT (CTB) and LT (LTB), paradoxically, have been shown to promote tolerance to heterologous antigens. CTB has been the most studied, especially as conjugates with antigens and it has been proposed as a strategy to prevent auto-immune and allergic diseases [13] and has been tested in clinical assays [14].

On the whole, these molecules (the whole toxins, their non toxic mutants or the B subunits) are potent immune modulators which are involved in complex interactions with the immune system, leading either to increase or decrease immune responses. 

Many studies have reported about the effects of the whole toxins or their mutants on different innate or adaptive immune cells that could explain the adjuvant effect (reviewed in [1,2,3,4,5,15]). However, the precise mechanism of action of these adjuvants has not been completely elucidated.

Different effects of the B subunit of these toxins on immune cells have also been reported but, conversely to the whole toxins and, because they potentiate tolerance and activation of regulatory T cells (Tregs) is a major mechanism involved in tolerance induction, the impact on Tregs has often been hypothesized, notably for CTB. 

The aim of this review is to make a point on what is known about Tregs and the cholera-like enterotoxins, including the whole molecules or their mutants as well as their subunits. 

First, the structures of these molecules will be described to underline similarities and differences which may impact the immunomodulatory properties, as well as the different regulatory T cell types. 

## 2. Structure of Cholera-Like Enterotoxins and Toxicity

***Whole toxins***. CT and LT are highly homologous (80% AA homology). They are made of a single A subunit which is associated to a non covalently linked pentameric ring of B subunit (A:B5). The A subunit is enzymatically active and consists of two chains A1 and A2 joined by a proteolytically sensitive peptide (Arg192) joined by a disulphide bond which has to be reduced for full enzymatic activity [5]. The main receptor for the B subunit of both CT and LT is GM1-ganglioside [16] that is found ubiquitously on the surface of mammalian cells, including enterocytes and immune cells, dendritic cells, macrophages, B and T cells. Other interactions have been reported which may have importance in immunological functions. Notably CTB and LTB also bind to GD1b-ganglioside but with a lower affinity and LTB, that binds to GM1 with a lower affinity than CTB, has been shown to have wider receptor specificity, as it binds notably to GM2 and asialo-GM1 [17]. The A1 fragment enters the cell cytosol and catalyses ADP-ribosylation of Gsa (GTP-binding protein family) which irreversibly activates adenylate cyclase and leads to an elevation in cyclic adenosine monophosphate (cAMP) levels. cAMP causes protein kinase A (PKA) to phosphorylate and open the cystic fibrosis transmembrane regulator (CFTR) Cl- channel, thus water and Cl- are flushed out causing diarrhea. The A2 fragment represents an adaptor molecule which interacts with the B subunit and may be involved in delivery of the A1 fragment into the correct cellular compartment and may also have a role in modulating the immunological effects. 

The type II subfamily (LT-II) includes two variants, LT-IIa and LT-IIb, that show high homology to CT and LT but recognize different ganglioside receptors, notably the B subunit of LT-IIb binds to Gd1a ganglioside [18].

***Mutants***. Site-directed mutagenesis has permitted the generation of mutants that have reduced toxicity. Mutants have been constructed to dissociate the enterotoxic effects from their adjuvant activity. Mutations in both the active and protease sites of CT and LT have been performed including peptide extension and amino acid deletion [1,3,5]. These mutants have been shown to possess adjuvant activity in animal models and some of them, the protease site mutant of LT, LT-R192G [8,9,10] and the enzymatic site mutant LTK63 [11,12] have been tested in humans. Another approach to detoxify CT has been proposed by Agren *et al.* [19] by linking the enzymatically active A subunit domain of the toxin to a synthetic analog of the *Staphylococcus aureus* protein A. The fusion protein devoid of the B subunit cannot bind to ganglioside receptors. CTA1-DD binds B cells, is non toxic despite enzymatic activity and has been shown to have adjuvant activity in animal models, notably after mucosal administration with chlamydial Major Outer Membrane Protein (MOMP), rotavirus VP6 chimeric protein and HIV1 envelope glycoproteins or as a fusion protein containing tandem repeats of the influenza matrix protein 2 (M2e) ectodomain epitope (CTA1-3M2e-DD) [20,21,22,23]. Of interest, two recent studies have reported that enzymatically inactive mutants fusion proteins of CTA1-DD containing either a type II collagen peptide CTA1R7K-COL-DD or an OVA peptide CTA1R7K-OVA-DD induced tolerance [24,25].

***B subunits***. To avoid toxicity, CTB and LTB have been examined for their ability to augment immune responses to heterologous antigens. Devoid of enzymatic activity, they are responsible for cell binding. However, CTB and LTB have been shown to promote tolerance to coadministered antigens. They have been used either coadministered with antigens or as antigen-conjugates, especially CTB. The binding of LT-IIb B subunit to GD1a ganglioside facilitates TLR2 recruitment and activation and probably confers on the molecule its specific proinflammatory potential and its adjuvant activity [18]. This observation highlights the importance of cell binding in immunomodulatory properties of these molecules.

## 3. Regulatory T cells (Figure 1)

Immunosuppression is an intrinsic property of the immune system and is partially mediated by T cells. The best defined T-cell population with immunosuppressive activity is the naturally activated subset of CD4^+^ T cells (nTregs), that constitutively express the interleukin (IL)-2Rα chain CD25 and Foxp3 (forkhead box p3) transcription factor, which controls the development and function of nTregs. These thymus-derived suppressor cells are present in normal individuals, contribute to the maintenance of self tolerance and protect from a variety of autoimmune diseases. They are also implicated in immune responses against pathogens [26,27]. nTregs exert suppressive effects via cell-cell contact or via cytokines, IL-10 and transforming growth factor β (TGFβ).

Regulatory T cells can also be induced from naïve peripheral CD4^+^CD25^-^ T cells after antigenic exposure. Induced Tregs include three subtypes, CD4^+^CD25^+^Foxp3^+^, Tr1 and Th3 regulatory T cells and their induction depends on the nature of the antigen, the context of antigen presentation, especially the type of dendritic cells, and the specificities of the tissue in which the antigen is delivered [28]. 

CD4^+^CD25^+^Foxp3^+^ iTregs have been generated *in vitro* and *in vivo* after TCR stimulation in the presence of IL-2 and TGFβ, under a variety of conditions. The gut-associated lymphoid tissue (GALT), which is a TGFβ-rich environment and contains a particular subset of dendritic cells producing retinoic acid, favors the development of CD4^+^CD25^+^Foxp3^+^ iTregs. Moreover, studies have shown that nTregs and iTregs work in synergy to protect the host [29]. 

Tr1 cells have been generated *in vitro* from naïve T cells in the presence of IL-10 and antigen. They have been shown to prevent colitis induced in SCID mice [30] and to be involved in suppressing immune responses towards food antigens. Classically, they produce high levels of IL-10 and no IL-4, but depending on experimental conditions, they can also secrete high amounts of TGFβ and IL-5 and low amounts of IFNγ and IL-2. Tr1 cells have no specific marker but are Foxp3^-^CD25^low^. They exert their suppressive effect via IL-10 and TGFβ [31].

Th3 cells are specifically triggered during oral tolerance. They express Foxp3 and secrete high amounts of TGFβ and some Th3 clones can produce IL-10 and IL-4. Moreover, they seem dependent on IL-4 (rather than IL-2) for growth. TGFβ plays a fundamental role in the normal intestine where it is abundant and TGFβ1 is an important mediator of epithelial cell differentiation and IgA class switching and it has immunosuppressive effects on lymphocytes. Moreover, TGFβ seems to be the link between the three induced Tregs mentioned above [32].

Besides CD4^+ ^Tregs, several types of CD8^+^ Tregs have also been reported [33]. As CD4^+^ Tregs, some CD8^+^ Tregs express Foxp3 and CD25, others exert a suppressor effect via TGFβ or via IL-10. CD8^+^ Tregs recognizing peptides associated with the MHC class Ib molecule Qa-1 and implicated in protection from experimental auto-immune encephalomyelitis (EAE) have also been reported. Moreover, in the intestine, intestinal intraepithelial lymphocytes (IEL) have been shown to have suppressive properties as TCRγδ IEL that secrete TGFβ and TCRαβ CD8αα IEL that secrete IL-10. Finally, CD8^+^ Tregs with suppressive properties are also present in the intestinal *lamina propria* [28].

**Figure 1:**
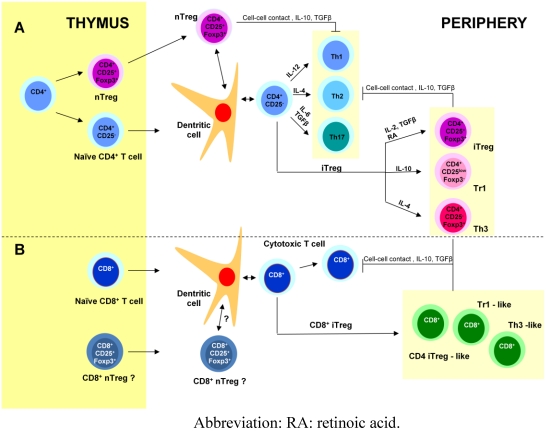
Generation of effector and regulatory T cells. Naïve CD4^+^ and CD8^+^ T cells as well as CD4^+^ and probably also CD8^+^ nTregs leave the thymus for the periphery where they colonize secondary lymphoid tissues and organs. A. Interaction between dendritic cells and naïve CD4^+^ T cells leads to different effector T cells (Th1, Th2 and Th17) and induced regulatory T cells (iTreg: Tr1, Th3 and CD4^+^CD25^+^Foxp3^+^ Tregs) depending on the cytokines produced in the micro-environment. Tregs modulate CD4^+^ and CD8^+^ effector responses via cell-cell contact or via cytokines, mainly IL-10 for Tr1 and TGFβ for Th3. B. As for CD4^+^ T cells, several induced CD8^+^ regulatory T cell subtypes have been reported: CD8^+^CD25^+^Foxp3^+^ T cells similar to CD4^+^ iTregs, Th3-like and Tr1-like CD8^+^ Tregs.

## 4. Adjuvanticity of Cholera-Like Enterotoxins

Many studies have reported on the mechanisms that could explain the adjuvant properties of CT and LT (reviewed in [1,2,3,4,5,15]). Effects on the epithelium (as a mechanical barrier and as a source of mediators) and on antigen presenting cells (APC) (dendritic cells (DC), macrophages and B cells) as well as the more recently described effect of LT-IIb B on TLR2 [18] are easy to correlate with adjuvanticity. Other effects such as CT or LT-induced cell death are more difficult to interpret because of a great complexity (different *in vivo*/*in vitro* results, importance of the state of maturation or activation of lymphocytes [34]). Finally, two recent studies suggested additional mechanisms for adjuvanticity. Lee *et al.* have reported that CT induced strong Th17-type responses through intranasal delivery [35], whereas CT was thought to induce anti-inflammatory responses (by promoting Th2 and inhibiting Th1 responses). Moreover, the CTB subunit may be responsible for the CT’s ability to induce Th17-dominated responses which is in contradiction with the tolerogenic role of CTB. Another study by Tamayo *et al.* [36] demonstrated that LT upregulates the expression of the glucocorticoid-induced TNFR-related protein (GITR) in CD4^+ ^T cells, that may contribute to adjuvanticity by acting as an activation-induced costimulatory signal in CD4^+^CD25^-^ cells. Of note, this effect was observed after systemic but not mucosal administration of LT, thus demonstrating that the mechanisms of adjuvanticity of LT are influenced by the route of administration.

## 5. Tolerogenicity of Cholera-Like Enterotoxins

The B subunit of both CT and LT has been shown to promote tolerance to heterologous antigens. Tolerogenicity induced by CTB or LTB may result from a direct depletion of effector T cells as the B subunits of CT and LT have been shown to induce CD4^+^ and CD8^+^ T cell apoptosis [5]. Another mechanism has been recently proposed by Wang *et al.* who showed that GM1 cross-linking by CTB resulted in the transient receptor potential canonical (TRPC) 5 channel activation which mediated Ca^2+^ influx in effector T cells and contributed to autoimmune suppression in an EAE model [37]. This effect mimics that of activated Tregs via Gal-1 on effector T cells. 

As a matter of fact, the most studied tolerogenic mechanism is the involvement of Tregs directly or via modulating APC.

## 6. Cholera-Like Enterotoxins and Tregs

As previously indicated, the generation and involvment of Tregs in immune modulation after administration of cholera-like molecules has been mainly hypothezised and studied with CTB or LTB coadministered with or conjugated to antigens, based on the tolerogenic properties of such Ag delivery systems. However, despite the great number of studies reporting on the effects of CT and LT on immune cells, few studies have looked at their impact on Tregs. 

### 6.1. B Subunits

#### 6.1.1. a-CTB

Many studies have reported that CTB induces tolerance against heterologous antigens and this strategy has been proposed to prevent auto-immune diseases such as EAE, diabetes, arthritis, uveitis or allergic diseases [38]. Some studies have examined the roles of Tregs; they are listed in Table 1 and Table 2. Most used Ag-CTB conjugates.

**Concerning auto-immune diseases** (Table 1), Tregs have been proposed to be involved in the protection from clinical disease after immunization with Ag-CTB conjugates by the oral or the nasal route as well as intraperitoneally, in animal models of EAE, diabetes and uveitis. All together, despite different doses and protocols used, these studies report the involvement of Treg cells, notably CD4^+^ Tregs, as demonstrated by their capacity to inhibit effector T cells *in vitro* and /or transfer experiments *in vivo*. Most of them underlined a decrease in Th1 and a shift to Th2 and/or Th3 T cells with IL-10, IL-4 and TGF production being increased. One study reported about CD4^+^CD45RC^+^RT6 memory regulatory T cells [38]. Of note, a dose- dependent effect was reported in two studies [39,40].

**Table 1:** CTB and regulatory T cells in auto-immune and inflammatory disease models.

**Model**	**Antigen**	**Protocol**	**Effects**	**Proposed Mechanism for Tolerance**	**Reference**
NOD mice Experimental diabetes	Hu insulin-CTB	Oral 1 dose 2–20 mg + transfer	Protection from clinical diabetes; Suppression of beta cell destruction	Protective T cells Non characterized	[42]
			Transfer of splenocytes induces protection		
NOD mice Experimental diabetes	Hu insulin-CTB	Oral 1 dose 10 mg + transfer	Increase in IL-4 (Th2); decrease in IFNg (Th1) in pancreatic LN + increase in TGFb in MLN	Ag specific CD4+ Tregs in the pancreas and draining LN Non characterized	[43]
			Transfer of CD4+ (but not CD8+) splenocytes induces protection		
NOD mice Experimental diabetes	Hu insulin- CTB	Nasal 1 dose 1 mg + transfer	Delays the incidence of diabetes ; dose-dependent effect; IL10 and TGFb increase in pancreas	CD4+ Tregs	[39]
			Transfer of CD4+ splenocytes induces protection	Non characterized	
				Dose-dependent effect	
H2d-RIP-LCMV-NP transgenic mice (LCMV induced diabetes)	Hu insulin-CTB or Porcine-insulin CTB	Oral 0.1-10 mg Biweekly (7 wks) + transfer	Transfer of splenocytes induces protection from diabetes and to bystander OVA antigen	CD4+ Tregs	[40]
			CD4 depletion abrogates protection	Non characterized	
			Dose- dependent effect (only intermetiate dosages are protective)	Dose- dependent effect	
NOD mice Experimental diabetes	CTB alone	IP 10 mg 3 times a week (4 weeks) + transfer	Decreases the development of clinical diabetes	Regulatory cells	[44]
			Transfer of splenocytes inhibits the adoptive transfer of diabetes by spleen cells from diabetic mice into irradiated NOD mice	Non characterized	
Rat EAE	MBP-CTB	Oral 50 mg 3 doses 4, 6 and 8 days after EAE induction transfer of MLN cells	Protection from clinical EAE; Decrease in CD4, CD8, IL-2R and MHC class II in spinal cord + Decrease in IFNg, IL-12, TNFa, MCP-1 and RANTES	Protective TGFb producing regulatory T cells	[45]
			Increase in TGFb	Non characterized	
			Transfer of MLN cells induces protection		
Rat experimental uveitis	Pept-HSP60- CTB	Oral 5 doses 15mg on alternate days Transfer of MLN cells	Increase in regulatory CD4+CD45RClowRT6+ subset of Th2 memory in MLN and spleen	Regulatory subset of memory cells	[38]
			Increase in IL-10, TGFb, decrease in IFNg and IL-12 in the MLN and the uveal tract	Shift from Th1 to Th2 and Th3 in the MLN and the uveal tract Non characterized	
			Prevention of uveitis		
			Transfer of MLN cells induces protection		
Apoe(-/-) mice atherosclerosis inflammatory disease model	Peptide apolipopt B-100-CTB	Nasal 15m g twice weekly12 weeks	Reduces aortic lesion size	Tr1 Tregs	[41]
			Induction of Tregs CD4+ IL-10+ (Tr1)TGF independent		

Abbreviations: EAE: experimental auto-immune encephalomyelitis; HSP : heat-shock protein; MBP: myelin basic protein; MLN: mesenteric lymph nodes.

Besides TGFβ producing Tregs, Tr1 Tregs have been recently reported in vaccination against atherosclerosis in mice [41]. Atherosclerosis is an inflammatory disease with auto-immune effectors contributing to disease progression. Intranasal immunization with apoB-CTB induced IL-10 producing Tr1 that inhibited effector responses to apoB-100 and reduced atherosclerosis. Moreover, these effects were TGFβ independent.

In summary, these studies show that Ag-CTB conjugates induce protective regulatory T cells in animal models of auto-immune or inflammatory diseases. Although not fully characterized TGFβ producing CD4^+^ Tregs (Th3 subtype?) have been most often reported in diabetes, EAE and uveitis, whereas in the atherosclerosis model, IL-10 producing Tr1 cells may be the major subtype involved. CD4^+^CD25^+^Foxp3^+^ T cells have not been reported nor CD8^+^ Tregs. Of note, variable doses and protocols of immunization have been used.

**Concerning allergic diseases** (Table 2), the administration of Ag conjugated to CTB has been shown to suppress IgE responses and to induce therapeutic effects [38]. The models used either OVA-CTB (dietary antigen) or the inhalant allergen Betv1a-CTB (major birch pollen allergen) as antigens, by the oral, the nasal or the sublingual route. Again, different doses and protocols were used. A role for Tregs has been reported. 

Sun *et al.* [46] showed that oral tolerance induced by OVA-CTB was associated with an increase in TGFβ in serum and an increase in both the frequency and suppressive capacity of Foxp3^+^CD25^+ ^Tregs together with the generation of both Foxp3^-^ and Foxp3^+^CD25^-^CD4^+^ Tregs. The coadministration of CT together with OVA-CTB abolished the increase in frequency of Tregs. The same authors reported that sublingual administration of OVA-CTB can also efficiently suppress peripheral effector T cell responses to OVA associated with a strong increase in serum TGFβ levels, Foxp3^+^CD25^+^CD4^+^ Treg cells and to a progressive depletion of effector T cells by apoptosis [47,48]. Effector T cell apoptosis was found to be critically dependent on CD25^+^ Treg cells but independent of IL-10 production. In contrast, Bublin *et al*. [49], using the Betv1a-CTB conjugate by the intranasal route in a model of allergic sensitization, showed that upregulation of Foxp3, IL-10 and TGFβ mRNA expression was detected in splenocytes but only after pretreatment with unconjugated allergen and not with the fusion molecule, indicating that antigen conjugation to a mucosal carrier modifies the immunomodulating properties of an antigen/allergen. Of note, opposite effects on IgE production, lymphocyte proliferation and cytokine production had already been reported by Wiedermann *et al.* [50] after intranasal administration of OVA-CTB and rBetv1a-CTB. Moreover, intranasaladministration prior to sensitization of unconjugated allergens alsoshowed contrasting effects: OVA could not significantlyinfluence antigen-specific antibody or cytokine production,whereas intranasal pretreatment with unconjugated Betv1a suppressedallergen-specific immune responses *in vivo* and *in vitro*. 

George-Chandy *et al.* (2006) [51] using a different Ag-conjugate, *i.e.*, a peptide from influenza haemagglutinin (HApep)-CTB conjugate, reported that CTB-induced oral tolerance could not be explained by CD25^+^ dependent regulatory activity, as oral administration of HApep-CTB to mice depleted of CD25^+^ cells still gave rise to systemic tolerance. 

**Table 2:** CTB and regulatory T cells in allergic disease models.

Model	Antigen	Protocol	Effects	Proposed Mechanism for Tolerance	Reference
BALB/c mice	OVA-CTB	Intragastric 3 doses (200 μg) at 2 days of interval	Increase in the frequency and suppressive activity of Ag-specific CD25^+^CD4^+^Foxp3^+^ Tregs (MLN, PP and spleen) abolished by the coadministration of CT (Table 4)	Foxp3^+^CD25^+^CD4^+^Tregs Foxp3^+^and Foxp3^-^CD25^-^CD4^+^Tregs	[46]
		Transfer of splenocytes and MLN cells	Generation of both Foxp3^+^ and Foxp3^-^CD25^-^CD4^+^		
			Increase in TGFβ (serum) CD25^+^ and CD25^-^ T cells suppress-effector Tcell proliferation *in vitro* - OVA-specific T cells and DTH after adoptive transfer		
BALB/c mice	OVA-CTB	Sublingual	Increase in Tregs in CLN MLN, spleen Increase in TGFβ (serum) Suppression of proliferative responses to OVA *in vitro* Suppression of OVA-specific DTH responses *in vivo* and T-cell proliferative responses in mice immunized SC with OVA + CFA	Foxp3^+^CD25^+^CD4^+^ Tregs	[47]
		One or 3 doses (40 μg)			
BALB/c mice	OVA-CTB	Sublingual	Development of OVA-specific Foxp3^+^CD25^+^CD4^+^ Tregs Suppression of peripheral T cell responses to OVA	CD4^+^CD25^+^Foxp3^+^ Tregs inhibit Teffector cell proliferation and induce Teffector cell apoptosis and depletion	[48]
		Adoptive transfer of OVA-specific TCR transgenic CD4^+^ T cells 3 doses (40 μg or 60 μg) at 2-day intervals.	Apoptosis of OVA-specific T effector cells in peripheral LN, dependent on CD25^+^ Treg cells		
BALB/c mice	Allergen Betv1a-CTB	Nasal 3 doses (20 μg) D0,D7,D14	Decrease in IgE, IL-5 Increase in IgG2a, IFNγ + local IgA , Th1 shift	Tolerance induction by the conjugate not associated with an increased expression of Foxp3, CTLA4 or the suppressive cytokines IL-10 and TGFβ in lymphocyte population of spleens or lungs	[49]
		allergic sensitization with the allergen	Upregulation of Foxp3, IL-10 and TGFβ mRNA in splenocytes after pretreatment with unconjugated allergen but not with the fusion molecule		

Abbreviations: CFA: complete Freund adjuvant; CLN: cervical lymph nodes; DTH: delayed-type hypersensibility; MLN: mesenteric lymph nodes; OVA: ovalbumin; PP: Peyer’s patches; SC: subcutaneously.

In conclusion, CD25^+^Foxp3^+^ as well as Foxp3^-^ and Foxp3^+^CD25^-^CD4^+^ Treg cells have been shown to be induced and to exert immunosuppressive effects after immunization with Ag-CTB in allergy experimental models. In auto-immune disease models, TGFβ producing Tregs (Th3?) have been the main Treg subtype reported. 

However, thetype of antigen or the nature of antigen-delivey system (coadministration vs Ag-conjugate) as well as the dose are major determinants that may lead to different, even opposite, effects

#### 6.1.2.b-LTB

LTB has been less studied than CTB (Table 3). In a model of collagen- induced arthritis (CIA), Luross *et al.* [52] report that ETxB (referred to LTB in this review) administered alone by the intranasal or the oral route, was effective in preventing collagen-induced arthritis. Disease protection could be transferred by CD4^+^ T cells from treated mice, an effect that was abrogated upon depletion of the CD25^+^ population. Of note, in this study, LTB was used alone and CTB failed to block CIA in the same conditions. The authors proposed that the inability of CTB, when used alone, to modulate CIA reveals critical differences between LTB and CTB, which probably relate to their disparate stabilities (*i.e.*, CTB is unstable as a pentamer below pH 3.9, and LTB is stable at pH 2.0) or the slightly wider receptor specificity of LTB.

Two studies reported that LTB exerts an immunomodulating effect that could be Treg-induced, as shown by the increase in TGFβ or IL-10, but together with an adjuvant effect. LTB abrogated oral tolerance to coadministered OVA in DO11.10 chimeric mice, resulting in a weak anti-OVA immune response, and an increase in TGFβ production [53]. In the same manner, the vaccination of latently HSV-1 (human herpes simplex virus type 1) infected mice with HSV-1 glycoproteins and LTB induced both systemic, eye and vaginal antibodies together with high levels of IL-10 and protection against both reactivation of latent virus and recurrent herpetic corneal epithelial disease and stromal disease [54].

Consistent with a balanced response, LTB has been reported by Raveney *et al.* [55] to protect mice from experimental auto-immune uveitis (EAU) development by inhibiting Th1 responses. Nevertheless, the resultant reduction in IFNγ responses by LTB does not affect infiltration or structural damage in established EAU, where Th17 responses predominate. However, this enhanced Th17 activity may be controlled directly by IL-10 or by increased levels of Treg activity as Th17 infiltration is not associated with increased symptoms.

Together, these results suggest that LTB like CTB induces CD25^+^ as well as IL-10 and/or TGFβ producing Tregs, but the balance response/tolerance may be different, either because of differences between the two subunits, notably in receptor binding but probably also because of the nature of the immunogens used (Ag-CTB conjugates *versus* coadministered Ag /LTB in the different studies).

**Table 3:** LTB and regulatory T cells.

**Model**	**Antigen**	**Protocol**	**Effects**	**Proposed Mechanism for Immuno-Modulation**	**Reference**
DBA/1 mice CIA	LTB alone	Nasal (100 μg) or intragastric (1 μg) transfer	Protection against CIA at the induction or 25 days later (not CTB)	CD4^+^CD25^+^ Tregs	[52]
			Decrease in IFNγ but not in IL-4 and IL-10		
			Transfer of CD4^+^ T cells induces protection abrogated upon depletion of the CD25^+^ population		
DO11.10 chimeric mice	LTB + OVA	Oral D1, 3, 5, and 7 (1 mg OVA+ 20 μg LTB)	Depressed IFNγ and enhanced TGFβ CTLA-4 up-regulation	Activated regulatory T cell populations as part of tolerance induction	[53]
Balb/c mice Latently infected by HSV-1 Therapeutic vaccination	LTB + HSV GP	Nasal 3 doses at 10-day intervals 10 μg HSV-1 glycoproteins + 20 μg LTB	Modulation of the Th1-dominated proinflammatory response induced upon infection	Tr1 Tregs	[54]
			Increase in IL-10 production by proliferating T cells from LN		
			Protection from HSV reactivation: decrease in incidence and severity of keratitis + reduction of virus spread + protection from encephalitis+ reduction in the incidence of recurrent herpetic corneal		
B10.RIII EAU	LTB alone	Nasal (50 μg) for 4 days, starting either 3 days before or 3 days after EAU induction	Preimmunization treatment protects from EAU decrease in Th1	IL-10 producing Tregs (Tr1)? modulate Th17 cells Critical importance of the dynamics of infiltration	[55]
			Treatment after induction does not protect despite decrease in IFNγ (Th1 decrease). Increases Th17 infiltration but not symptoms		

Abbreviations: CIA: collagen-induced arthritis; EAU: experimental auto-immune uveitis; GP: glycoprotein; HSV-1: human herpes simplex virus type 1.

### 6.2. A Subunit: CTA1-DD and Mutant (Table 4)

CTA1-DD lacks the B subunit as it comprises the A subunit of cholera toxin linked to a synthetic analog of *S. aureus* protein A. Whereas CTA1-DD has been shown to have adjuvant activity, a mutant of CTA1-DD, CTA1R7K-DD, that is devoid of ADP -ribosyl transferase activity has been reported to induce tolerance when used by the IN route as a fusion protein including either a type II collagen peptide or an OVA peptide [24,25]. The mechanism behind the tolerance to collagen induced arthritis appears to be mediated by peptide-specific regulatory T cells induced by mucosal exposure to the peptide containing CTA1R7K-COL-DD vector. Moreover, using a fusion protein CTA1R7K-OVA-DD, Hasselberg *et al.* demonstrated that ADP-ribosylation controls the outcome of tolerance or active effector T cell immunity as a single point mutation, resulting in lack of enzymatic activity, promoted peptide-specific tolerance in TCR transgenic CD4^+^ T cells following a single intranasal treatment [24].

**Table 4:** CTA1-DD mutant and regulatory T cells.

**Model**	**Protocol**	**Effects**	**Proposed Mechanism for Tolerance**	**Reference**
DBA-1 mice	Nasal	Protection against CIA	Peptide specific induced Tregs Tr1?	[25]
	3 doses 5 μg on D5, 6, 7	Lower serum anti-collagen antibodies		
	CTA1R7K-COL-DD	Decrease in IL-6, IL-17, IFNγ		
	after the collagen boost/induction of CIA.	Increase in IL-10 in serum and at the T cell level.		
BALB/c mice	Nasal	Induction of long-lived specific tolerance to OVA,	Induction of CD4^+^CD25^-^Foxp3^-^ Tr1 cells producing IL-10	[24]
	CTA1R7K-OVA-DD	Induction of IL-10 Tregs Dependent on enzymatic activity		

Abbreviations: CIA: collagen-induced arthritis.

### 6.3. Whole Toxins and Mutants (Table 5)

Three studies have hypothesized an effect of CT on Tregs as a mechanism of adjuvanticity (Table 5). As early as 1995, Elson *et al.* [56] showed a profound reduction in CD8^+^ IEL shortly after a mucosal exposure of mice to CT. They proposed that one of the mechanisms of CT’s mucosal effects *in vivo* was the alteration of the regulatory T cell environment in the GALT . Flach *et al*. [57] confirmed in rats that intragastric CT challenge rapidly affects CD8^+^ IEL within the intestinal mucosa, but suggested a cell migration rather than cell death. In both studies, the immunomodulating role of CD8^+^ IEL depletion has been suggested but not demonstrated. 

Sun *et al.* [46] showed an effect of CT on CD4^+^ Tregs. Indeed, they found that the coadministration of even very small amounts of CT together with the OVA/CTB conjugate effectively prevented the induction of CD25^+^CD4^+^ suppressor T cells. Furthermore, CT also inhibited the normal suppressive function and Foxp3 gene expression of the mucosal CD25^+^ Treg cells in the OVA/CTB-treated mice in comparison with PBS-fed mice. However, they observed only a relatively small effect of CT given after, rather than together, with a tolerizing OVA/CTB feeding regimen. 

At the opposite, Lavelle *et al.* [58,59] showed that parenteral immunization (footpad) of mice with keyhole limpet haemocyanin (KLH) in the presence of CT promoted the induction of Tr1 cells specific for KLH, that suppressed IFNγ production by Th1 cells as a consequence of an effect of CT on DC maturation and cytokine production (induction of IL-10 and inhibition of IL-12). The Tr1 subpopulation was further characterized as producing IL-10 but not IL-4 and as acting by a cell independent contact mechanism [58,59]. Although the route of administration may be an important factor to consider, this study suggests that CT promotes Treg induction. The results we obtained with a protease site mutant of LT, LT-R192G, showed that it decreases CD4^+^CD25^+^Foxp3^+^ T cells *in vitro* during a first contact (probable nTregs). However, consistent with the results of Lavelle *et al.*, LT-R192G induces specific CD4^+^CD25^+^Foxp3^+^ T cells that can be recalled *in vitro* in the presence of LT-R192G (Foxp3 upregulation) and also promotes the induction of Ag (rotavirus-like particles)-specific CD4^+^CD25^+^Foxp3^+^ T cells [60] that can be recalled *in vitro* in the presence of Ag (upregulation of both CD25 and Foxp3). Thus, the whole molecules, despite adjuvant properties, may also induce or promote Tregs.

### 6.4. Mechanisms

Different mechanisms, that are not exclusive, have been suggested to explain how these toxins may exert their effects on Tregs. They may affect either Treg number or Treg function by 1) decreasing Tregs 2) modulating APC thus leading to Treg induction or inhibition 3) modulating Treg interaction with target cells (cell-cell contact) 4) increasing the production of soluble suppressive factors such as IL-10 and TGFβ. 

***Treg decrease***. The whole toxins or their mutants that have an adjuvant effect may specifically decrease Treg number. However, although CT and LT have been shown to induce a depletion of CD8^+ ^IEL [56,57] and CD4^+^ and CD8^+^ lymphocytes by *in vivo* apoptosis [34], respectively, a specific decrease of Tregs *in vivo* has not been demonstrated. We have shown a decrease of CD4^+ ^CD25^+^Foxp3^+^ T cells during a first *in vitro* contact with the mutant LT-R192G but not during an *in vitro* recall, suggesting a probable specific effect on nTregs but not on Tregs induced in immunized mice [60]. However, no significant decrease has been observed *in vivo* after a first immunization (*data not published*). 

***APC modulation***.Dendritic cells (DC) play a determinant role in the orientation of immune responses. *Whole toxins* have been shown to have many effects on DC (reviewed in [1,2,3]), they notably upregulate class II MHC as well as costimulatory molecules, leading to improved capacity to present antigens. Negri *et al.* [61] also reported that CT and LT, but not their non toxic derivates, improve the antigen-presenting cell function of human B lymphocytes *in vitro*. However, whether these effects render effector T cells unresponsive to suppression and/or lead to Treg inhibition has not been demonstrated. Similarly, GITR, which is upregulated by systemic administration of LT [36], may also help target cells to evade suppression or inhibit the suppressive capacity of Tregs, but as for costimulatory molecules, a direct impact of LT on Tregs has not been demonstrated. Anjuere *et al.* [62] found that oral administration of CT to mice resulted in a marked increase in PP and MLN of CD11c^+^CD8^int ^DC with potent immunological antigen presentation, together with inhibition of the normal development of tolerogenic CD11c^+^CD8^+^B220^+^ (plasmacytoid) DC in response to CTB administration, but again, inhibition of Tregs was not reported. Moreover, Lavelle *et al.* have reported that CT promotes the induction of T cells with regulatory activity (Tr1) by inducing IL-10 production by DC and inhibiting IL-12 [59].

**Table 5:** Whole toxins and mutants and regulatory T cells.

**Model**	**Protocol**	**Route**	**Effects**	**Conclusion**	**Reference**
C57B1/6 or CB6F1 mice	CT (10 μg) or CTB (100 μg) + transfer of splenocytes from donors fed with CT+ KLH	Intragastric	IEL CD8^+^ depletion in the group receiving CT	Abrogation of suppressor T cell function *in vivo* by mucosal CT	[56]
			T cell suppression of both secretory IgA and plasma IgG anti-KLH after KLH feeding and its abrogation by CT	Tregs non characterized	
Rat	CT (100 μg) or CTB (62 μg)	Intragastric	CT induces a transient depletion of jejunal CD8 (IEL)	Disturbance of the gut homeostasis	[57]
				Tregs non characterized	
Balb/c mice	OVA-CTB 3 doses (200 μg) at 2 days of interval +/− CT (4 μg)	Intragastric	CT abrogates the increase in the frequency and suppressive activity of Ag-specific CD25^+^CD4^+^Foxp3^+^Tregs (MLN, PP and spleen) induced by CTB	CT prevents the induction of CD25^+^CD4^+^ suppressor T cells by OVA-CTB	[46] Cf Table 2
				CT also inhibits the normal suppressive function and Foxp3 gene expression of the mucosal CD25^+^ Treg cells in the OVA/CTB-treated mice	
BALB/c, C3H/HeN, and C3H/HeJ mice	D0: KLH (10 μg) or KLH (10 μg) and CT (1.0 μg) D7: KLH (20 μg) Generation of Ag specific Tcell lines and clones Cocultures of DC + LPS + CT	SC footpad	Induction of Th2 and Tr1 cells (specific for KLH); Inhibition of IFNγ production	CT promotes the induction of Tr1 cells specific for bystander Ag	[59]
			DC + LPS + CT: CT induces maturation of DC, induces IL-10, and inhibits IL-12 production	Effect on DC	
Balb /c mice	D0: LT-R192G (10 μg) + rotavirus VLP (10 μg) D14: *in vitro* restimulation with Ag, LT-R192G or both	Intrarectal	LT-R192G decreases *in vitro* CD4^+^CD25^+^Foxp3^+^ from non immunized mice (nTregs?)	Effect of LT-R192G on CD4^+^CD25^+^Foxp3^+ ^Tregs, different between a first contact and a recall	[60]
			LT-R192G induces specific CD4^+^CD25^+^Foxp3^+^ and promotes the induction of CD4^+^CD25^+^Foxp3^+^ specific for the Ag		

Abbreviations: DC: dendritic cells; IEL: intra-epithelial lymphocytes; KLH: keyhole limpet hemocyanin; VLP: viral like particles.

Conversely to CT, CTB is expected to modulate APC leading to Treg induction. Indeed, CTB has been reported to induce regulatory Tr1 cells by preventing human DC maturation on the basis that it partially prevents LPS induced maturation of monocyte derived dendritic cells (MDDC), decreases their IL-12 production and, in cocultures with T cells promotes IL-10 [63]. Besides DC, B cells may play an important and probably underestimated role in promoting Tregs. Sun *et al.*, [64] using µMT−/− B cell-deficient mice, showed the importance of B cells for the induction of Ag-specific Foxp3^+^ Treg cells and oral tolerance. They proposed that the high-affinity binding of CTB-Ag complexes to B cell (or other APC) rafts via the GM1 receptor and the exceptional persistence of such complexes on the cell surface may be important both for inducing tolerogenic effects in the B cells and possibly also immune synapse formation of B cells with Ag-specific T cells promoting the development and expansion of Treg cells. Of note, Thiam *et al.* [60] and Ogier *et al.* [65] observed a massive expansion of Ag specific B cells after mucosal immunization with rotavirus–like particles and LT-R192G, after a prime but not after a boost; most of them were non conventional B1-a cells expressing CD5. We hypothesized that this massive expansion may be suppressed by Tregs induced in the presence of the adjuvant. We found that CD4^+^CD25^+^Foxp3^+^ specific for both the antigen and the adjuvant were induced after a prime, but the role of B cells in inducing these cells remains to be determined. 

In summary, as expected, CTB has been reported to modulate APC, either DC or B cells, to induce Tregs, but CT has also been shown to modulate APC to induce Tr1 cells. 

### 6.5. Direct effect on Treg-target cell interaction

The cholera-like enterotoxins may impact on Treg-target cell interaction either by modulating delivery of suppressive factors via gap junctions including cAMP, by inducing membrane-bound suppressive cytokines such as TGFβ or by increasing CTLA-4 expression by Tregs.

#### 6.5.1. cAMP

Vendetti *et al*. [66] reported that CT-pretreated CD4^+^ T lymphocytes can exert regulatory functions by inhibiting the proliferation of autologous PBMC through the release of extracellular cAMP and that the cyclic nucleotide acts as a primary messenger, which could play a biological role in the modulation of immune responses. This result is consistent with results published in 2007 by Bopp *et al.* [67] which showed that Tregs can increase cAMP levels in the target cells by delivery via gap junctions.

#### 6.5.2. TGFβ

TGFβ has been shown to be induced by Ag-CTB conjugates in many studies (Table 1 and Table 2). TGFβ is an immunosuppressive cytokine that can also contribute to cell-contact suppression when bound to membrane [68], however the importance of this mechanism in tolerogenicity induced by CTB has not been demonstrated.

#### 6.5.3. CTLA-4

CT has been shown to up-regulate the expression of the inhibitory molecule CTLA-4 in naïve, effector and memory resting CD4^+^ T cells and in resting CD8^+^ T lymphocytes [66]. Tregs constitutively express CTLA-4 that has been shown to have a specific role in the function of CD4^+^CD25^+^ Tregs [69]. One can hypothesize that CT may also up-regulate CTLA-4 expression on Tregs thus modulating their function. 

In summary, although these 3 mediators that may improve Treg function are upregulated by CT and/or LT, an impact on Tregs has been demonstrated only for cAMP. Of note, cAMP increase is correlated with the enzymatic activity and thus only the whole toxins may exert an immunosuppressive effect via cAMP.

## 7. Conclusions

Both the whole toxins and the B subunits have been shown to modulate Tregs. Moreover, different mechanisms may account for their effects and, except for the mechanism involving cAMP, it is difficult to associate one mechanism with a type of molecule, *i.e.*, whole toxin or mutant vs B subunit, thus confirming the great complexity of these molecules and the difficulty to categorize them into adjuvants on the one hand and tolerance inducers on the other hand. However, the recent report by Hasselberg *et al*. showing that ADP-ribosylation controls the outcome of tolerance or active effector T cell immunity to an internal peptide p323–339 from OVA inserted into the cholera toxin (CT)-derived CTA1-OVA-DD [24] demonstrates that it is possible to decipher at the molecular level the critical determinants that impact on the orientation of immune responses. 

Nevertheless, the variability of the effects depending on the type of antigen, the dose, the route and the time of administration (prime/boost or prevention/treatment) makes it imperative to test these molecules in different models.

As these molecules are potent immunomodulating agents with therapeutic potential in humans, future research to improve understanding of their effects is necessary.

## References

[1] Freytag L.C., Clements J.D. (2005). Mucosal adjuvants. Vaccine.

[2] Cox E., Verdonck F., Vanrompay D., Goddeeris B. (2006). Adjuvants modulating mucosal immune responses or directing systemic responses towards the mucosa. Vet. Res..

[3] Sanchez J., Holmgren J. (2008). Cholera toxin structure, gene regulation and pathophysiological and immunological aspects. Cell Mol. Life Sci..

[4] Williams N.A., Hirst T.R., Nashar T.O. (1999). Immune modulation by the cholera-like enterotoxins: From adjuvant to therapeutic. Immunol. Today.

[5] Rappuoli R., Pizza M., Douce G., Dougan G. (1999). Structure and mucosal adjuvanticity of cholera and Escherichia coli heat-labile enterotoxins. Immunol. Today.

[6] Pickett C.L., Twiddy E.M., Belisle B.W., Holmes R.K. (1986). Cloning of genes that encode a new heat-labile enterotoxin of Escherichia coli. J. Bacteriol..

[7] Holmes R.K., Twiddy E.M., Pickett C.L. (1986). Purification and characterization of type II heat-labile enterotoxin of Escherichia coli. Infect. Immun..

[8] Kotloff K.L., Sztein M.B., Wasserman S.S., Losonsky G.A., DiLorenzo S.C., Walker R.I. (2001). Safety and immunogenicity of oral inactivated whole-cell Helicobacter pylori vaccine with adjuvant among volunteers with or without subclinical infection. Infect Immun..

[9] Lapa J.A., Sincock S.A., Ananthakrishnan M., Porter C.K., Cassels F.J., Brinkley C., Hall E.R., van Hamont J., Gramling J.D., Carpenter C.M., Baqar S., Tribble D.R. (2008). Randomized clinical trial assessing the safety and immunogenicity of oral microencapsulated enterotoxigenic Escherichia coli surface antigen 6 with or without heat-labile enterotoxin with mutation R192G. Clin. Vaccine Immunol..

[10] Lemere C.A. (2009). Developing novel immunogens for a safe and effective Alzheimer's disease vaccine. Prog. Brain Res..

[11] Lewis D.J., Huo Z., Barnett S., Kromann I., Giemza R., Galiza E., Woodrow M., Thierry-Carstensen B., Andersen P., Novicki D., Del Giudice G., Rappuoli R. (2009). Transient facial nerve paralysis (Bell's palsy) following intranasal delivery of a genetically detoxified mutant of Escherichia coli heat labile toxin. PLoS One.

[12] Peppoloni S., Ruggiero P., Contorni M., Morandi M., Pizza M., Rappuoli R., Podda A., Del Giudice G. (2003). Mutants of the Escherichia coli heat-labile enterotoxin as safe and strong adjuvants for intranasal delivery of vaccines. Expert Rev. Vaccines.

[13] Sun J.B., Czerkinsky C., Holmgren J. (2010). Mucosally induced immunological tolerance, regulatory T cells and the adjuvant effect by cholera toxin B subunit. Scand J. Immunol..

[14] Stanford M., Whittall T., Bergmeier L.A., Lindblad M., Lundin S., Shinnick T., Mizushima Y., Holmgren J., Lehner T. (2004). Oral tolerization with peptide 336-351 linked to cholera toxin B subunit in preventing relapses of uveitis in Behcet's disease. Clin. Exp. Immunol..

[15] Mestecky J., Russell M.W., Elson C.O. (2007). Perspectives on mucosal vaccines: Is mucosal tolerance a barrier?. J. Immunol..

[16] Holmgren J. (1973). Comparison of the tissue receptors for Vibrio cholerae and Escherichia coli enterotoxins by means of gangliosides and natural cholera toxoid. Infect. Immun..

[17] Fukuta S., Magnani J.L., Twiddy E.M., Holmes R.K., Ginsburg V. (1988). Comparison of the carbohydrate-binding specificities of cholera toxin and Escherichia coli heat-labile enterotoxins LTh-I, LT-IIa, and LT-IIb. Infect Immun..

[18] Liang S., Hosur K.B., Lu S., Nawar H.F., Weber B.R., Tapping R.I., Connell T.D., Hajishengallis G. (2009). Mapping of a microbial protein domain involved in binding and activation of the TLR2/TLR1 heterodimer. J. Immunol..

[19] Agren L.C., Ekman L., Lowenadler B., Nedrud J.G., Lycke N.Y. (1999). Adjuvanticity of the cholera toxin A1-based gene fusion protein, CTA1-DD, is critically dependent on the ADP-ribosyltransferase and Ig-binding activity. J. Immunol..

[20] Cunningham K.A., Carey A.J., Lycke N., Timms P., Beagley K.W. (2009). CTA1-DD is an effective adjuvant for targeting anti-chlamydial immunity to the murine genital mucosa. J. Reprod. Immunol..

[21] McNeal M.M., Basu M., Bean J.A., Clements J.D., Lycke N.Y., Ramne A., Lowenadler B., Choi A.H., Ward R.L. (2007). Intrarectal immunization of mice with VP6 and either LT(R192G) or CTA1-DD as adjuvant protects against fecal rotavirus shedding after EDIM challenge. Vaccine.

[22] Sundling C., Schon K., Morner A., Forsell M.N., Wyatt R.T., Thorstensson R., Karlsson Hedestam G.B., Lycke N.Y. (2008). CTA1-DD adjuvant promotes strong immunity against human immunodeficiency virus type 1 envelope glycoproteins following mucosal immunization. J. Gen. Virol..

[23] Eliasson D.G., El Bakkouri K., Schon K., Ramne A., Festjens E., Lowenadler B., Fiers W., Saelens X., Lycke N. (2008). CTA1-M2e-DD: A novel mucosal adjuvant targeted influenza vaccine. Vaccine.

[24] Hasselberg A., Ekman L., Yrlid L.F., Schon K., Lycke N.Y. (2010). ADP-ribosylation controls the outcome of tolerance or enhanced priming following mucosal immunization. J. Immunol..

[25] Hasselberg A., Schon K., Tarkowski A., Lycke N. (2009). Role of CTA1R7K-COL-DD as a novel therapeutic mucosal tolerance-inducing vector for treatment of collagen-induced arthritis. Arthritis Rheum..

[26] Josefowicz S.Z., Rudensky A. (2009). Control of regulatory T cell lineage commitment and maintenance. Immunity.

[27] Sakaguchi S., Yamaguchi T., Nomura T., Ono M. (2008). Regulatory T cells and immune tolerance. Cell.

[28] Saurer L., Mueller C. (2009). T cell-mediated immunoregulation in the gastrointestinal tract. Allergy.

[29] Curotto de Lafaille M.A., Lafaille J.J. (2009). Natural and adaptive foxp3+ regulatory T cells: More of the same or a division of labor?. Immunity.

[30] Groux H., O'Garra A., Bigler M., Rouleau M., Antonenko S., de Vries J.E., Roncarolo M.G. (1997). A CD4+ T-cell subset inhibits antigen-specific T-cell responses and prevents colitis. Nature.

[31] Roncarolo M.G., Gregori S., Battaglia M., Bacchetta R., Fleischhauer K., Levings M.K. (2006). Interleukin-10-secreting type 1 regulatory T cells in rodents and humans. Immunol. Rev..

[32] Faria A.M., Weiner H.L. (2005). Oral tolerance. Immunol. Rev..

[33] Shevach E.M. (2006). From vanilla to 28 flavors: Multiple varieties of T regulatory cells. Immunity.

[34] Tamayo E., Postigo J., Del Giudice G., Rappuoli R., Benito A., Yagita H., Merino R., Merino J. (2009). Involvement of the intrinsic and extrinsic cell-death pathways in the induction of apoptosis of mature lymphocytes by the Escherichia coli heat-labile enterotoxin. Eur. J. Immunol..

[35] Lee J.B., Jang J.E., Song M.K., Chang J. (2009). Intranasal delivery of cholera toxin induces th17-dominated T-cell response to bystander antigens. PLoS One.

[36] Tamayo E., Postigo J., Gonzalez J., Fernandez-Rey M., Iglesias M., Santiuste I., Riccardi C., Rappuoli R., Del Giudice G., Merino R., Merino J. (2009). GITR contributes to the systemic adjuvanticity of the Escherichia coli heat-labile enterotoxin. Eur. J. Immunol..

[37] Wang J., Lu Z.H., Gabius H.J., Rohowsky-Kochan C., Ledeen R.W., Wu G. (2009). Cross-linking of GM1 ganglioside by galectin-1 mediates regulatory T cell activity involving TRPC5 channel activation: Possible role in suppressing experimental autoimmune encephalomyelitis. J. Immunol..

[38] Phipps P.A., Stanford M.R., Sun J.B., Xiao B.G., Holmgren J., Shinnick T., Hasan A., Mizushima Y., Lehner T. (2003). Prevention of mucosally induced uveitis with a HSP60-derived peptide linked to cholera toxin B subunit. Eur. J. Immunol..

[39] Aspord C., Thivolet C. (2002). Nasal administration of CTB-insulin induces active tolerance against autoimmune diabetes in non-obese diabetic (NOD) mice. Clin. Exp. Immunol..

[40] Petersen J.S., Bregenholt S., Apostolopolous V., Homann D., Wolfe T., Hughes A., De Jongh K., Wang M., Dyrberg T., Von Herrath M.G. (2003). Coupling of oral human or porcine insulin to the B subunit of cholera toxin (CTB) overcomes critical antigenic differences for prevention of type I diabetes. Clin. Exp. Immunol..

[41] Klingenberg R., Lebens M., Hermansson A., Fredrikson G.N., Strodthoff D., Rudling M., Ketelhuth D.F., Gerdes N., Holmgren J., Nilsson J., Hansson G.K. (2010). Intranasal Immunization With an Apolipoprotein B-100 Fusion Protein Induces Antigen-Specific Regulatory T Cells and Reduces Atherosclerosis. Arterioscler Thromb. Vasc. Biol..

[42] Bergerot I., Ploix C., Petersen J., Moulin V., Rask C., Fabien N., Lindblad M., Mayer A., Czerkinsky C., Holmgren J., Thivolet C. (1997). A cholera toxoid-insulin conjugate as an oral vaccine against spontaneous autoimmune diabetes. Proc. Natl. Acad. Sci. USA.

[43] Ploix C., Bergerot I., Durand A., Czerkinsky C., Holmgren J., Thivolet C. (1999). Oral administration of cholera toxin B-insulin conjugates protects NOD mice from autoimmune diabetes by inducing CD4+ regulatory T-cells. Diabetes.

[44] Sobel D.O., Yankelevich B., Goyal D., Nelson D., Mazumder A. (1998). The B-subunit of cholera toxin induces immunoregulatory cells and prevents diabetes in the NOD mouse. Diabetes.

[45] Sun J.B., Xiao B.G., Lindblad M., Li B.L., Link H., Czerkinsky C., Holmgren J. (2000). Oral administration of cholera toxin B subunit conjugated to myelin basic protein protects against experimental autoimmune encephalomyelitis by inducing transforming growth factor-beta-secreting cells and suppressing chemokine expression. Int. Immunol..

[46] Sun J.B., Raghavan S., Sjoling A., Lundin S., Holmgren J. (2006). Oral tolerance induction with antigen conjugated to cholera toxin B subunit generates both Foxp3+CD25+ and Foxp3-CD25- CD4+ regulatory T cells. J. Immunol..

[47] Sun J.B., Cuburu N., Blomquist M., Li B.L., Czerkinsky C., Holmgren J. (2006). Sublingual tolerance induction with antigen conjugated to cholera toxin B subunit induces Foxp3+CD25+CD4+ regulatory T cells and suppresses delayed-type hypersensitivity reactions. Scand. J. Immunol..

[48] Sun J.B., Czerkinsky C., Holmgren J. (2007). Sublingual 'oral tolerance' induction with antigen conjugated to cholera toxin B subunit generates regulatory T cells that induce apoptosis and depletion of effector T cells. Scand. J. Immunol..

[49] Bublin M., Hoflehner E., Wagner B., Radauer C., Wagner S., Hufnagl K., Allwardt D., Kundi M., Scheiner O., Wiedermann U., Breiteneder H. (2007). Use of a genetic cholera toxin B subunit/allergen fusion molecule as mucosal delivery system with immunosuppressive activity against Th2 immune responses. Vaccine.

[50] Wiedermann U., Jahn-Schmid B., Lindblad M., Rask C., Holmgren J., Kraft D., Ebner C. (1999). Suppressive *versus* stimulatory effects of allergen/cholera toxoid (CTB) conjugates depending on the nature of the allergen in a murine model of type I allergy. Int. Immunol..

[51] George Chandy A., Hultkrantz S., Raghavan S., Czerkinsky C., Lebens M., Telemo E., Holmgren J. (2006). Oral tolerance induction by mucosal administration of cholera toxin B-coupled antigen involves T-cell proliferation *in vivo* and is not affected by depletion of CD25+ T cells. Immunology.

[52] Luross J.A., Heaton T., Hirst T.R., Day M.J., Williams N.A. (2002). Escherichia coli heat-labile enterotoxin B subunit prevents autoimmune arthritis through induction of regulatory CD4+ T cells. Arthritis Rheum..

[53] Plant A., Williams R., Jackson M.E., Williams N.A. (2003). The B subunit of Escherichia coli heat labile enterotoxin abrogates oral tolerance, promoting predominantly Th2-type immune responses. Eur. J. Immunol..

[54] Richards C.M., Case R., Hirst T.R., Hill T.J., Williams N.A. (2003). Protection against recurrent ocular herpes simplex virus type 1 disease after therapeutic vaccination of latently infected mice. J. Virol..

[55] Raveney B.J., Richards C., Aknin M.L., Copland D.A., Burton B.R., Kerr E., Nicholson L.B., Williams N.A., Dick A.D. (2008). The B subunit of Escherichia coli heat-labile enterotoxin inhibits Th1 but not Th17 cell responses in established experimental autoimmune uveoretinitis. Invest. Ophthalmol. Vis. Sci..

[56] Elson C.O., Holland S.P., Dertzbaugh M.T., Cuff C.F., Anderson A.O. (1995). Morphologic and functional alterations of mucosal T cells by cholera toxin and its B subunit. J. Immunol..

[57] Flach C.F., Lange S., Jennische E., Lonnroth I., Holmgren J. (2005). Cholera toxin induces a transient depletion of CD8+ intraepithelial lymphocytes in the rat small intestine as detected by microarray and immunohistochemistry. Infect. Immun..

[58] Lavelle E.C., Jarnicki A., McNeela E., Armstrong M.E., Higgins S.C., Leavy O., Mills K.H. (2004). Effects of cholera toxin on innate and adaptive immunity and its application as an immunomodulatory agent. J. Leukoc. Biol..

[59] Lavelle E.C., McNeela E., Armstrong M.E., Leavy O., Higgins S.C., Mills K.H. (2003). Cholera toxin promotes the induction of regulatory T cells specific for bystander antigens by modulating dendritic cell activation. J. Immunol..

[60] Thiam F., di Martino C., Bon F., Charpilienne A., Cachia C., Poncet D., Clements J.D., Basset C., Kohli E. (2010). Unexpected modulation of recall B and T cell responses after immunization with rotavirus- like particles in the presence of LT-R192G. Toxins.

[61] Negri D.R., Pinto D., Vendetti S., Patrizio M., Sanchez M., Riccomi A., Ruggiero P., Del Giudice G., De Magistris M.T. (2009). Cholera toxin and Escherichia coli heat-labile enterotoxin, but not their nontoxic counterparts, improve the antigen-presenting cell function of human B lymphocyte. Infect. Immun..

[62] Anjuere F., Luci C., Lebens M., Rousseau D., Hervouet C., Milon G., Holmgren J., Ardavin C., Czerkinsky C. (2004). *In vivo* adjuvant-induced mobilization and maturation of gut dendritic cells after oral administration of cholera toxin. J. Immunol..

[63] D'Ambrosio A., Colucci M., Pugliese O., Quintieri F., Boirivant M. (2008). Cholera toxin B subunit promotes the induction of regulatory T cells by preventing human dendritic cell maturation. J. Leukoc. Biol..

[64] Sun J.B., Flach C.F., Czerkinsky C., Holmgren J. (2008). B lymphocytes promote expansion of regulatory T cells in oral tolerance: Powerful induction by antigen coupled to cholera toxin B subunit. J. Immunol..

[65] Ogier A., Franco M.A., Charpilienne A., Cohen J., Pothier P., Kohli E. (2005). Distribution and phenotype of murine rotavirus-specific B cells induced by intranasal immunization with 2/6 virus-like particles. Eur. J. Immunol..

[66] Vendetti S., Patrizio M., Riccomi A., De Magistris M.T. (2006). Human CD4+ T lymphocytes with increased intracellular cAMP levels exert regulatory functions by releasing extracellular cAMP. J. Leukoc. Biol..

[67] Bopp T., Becker C., Klein M., Klein-Hessling S., Palmetshofer A., Serfling E., Heib V., Becker M., Kubach J., Schmitt S., Stoll S., Schild H., Staege M.S., Stassen M., Jonuleit H., Schmitt E. (2007). Cyclic adenosine monophosphate is a key component of regulatory T cell-mediated suppression. J. Exp. Med..

[68] Nakamura K., Kitani A., Strober W. (2001). Cell contact-dependent immunosuppression by CD4(+)CD25(+) regulatory T cells is mediated by cell surface-bound transforming growth factor beta. J. Exp. Med..

[69] Read S., Greenwald R., Izcue A., Robinson N., Mandelbrot D., Francisco L., Sharpe A.H., Powrie F. (2006). Blockade of CTLA-4 on CD4+CD25+ regulatory T cells abrogates their function *in vivo*. J. Immunol..

